# Knowledge and Attitude of Medical Students in Lebanon Towards Disaster Medicine

**DOI:** 10.3389/ijph.2025.1608095

**Published:** 2025-07-28

**Authors:** Batoul Issam Abbas, Hasan Houssein Abbass, Azza Ali Ali Hasan, Abdul Hamid Mohamad Alwan, Nourhan Hussein Azzam, Joudy Hussien Al Sahmarani, Ilham Sleiman Hassan, Noama Wassek El Husseini, Bahaa Wadih Bou Dargham

**Affiliations:** ^1^Faculty of Medicine, Beirut Arab University, Beirut, Lebanon; ^2^ Hammoud Hospital University Medical Center, Saida, Lebanon

**Keywords:** disaster medicine, medical students, knowledge, attitude, Lebanon

## Abstract

**Objectives:**

This study aims to assess the knowledge and attitude of medical students in Lebanon towards disaster medicine.

**Methods:**

An exploratory online cross-sectional survey was conducted on 388 medical students from different educational levels and universities in Lebanon. The data was analyzed using the Statistical Package for Social Sciences (SPSS), considering a p-value of <0.05 as significant.

**Results:**

The participants had a mean knowledge score of 12.19 ± 4.04 out of 25 questions. Those who received a disaster medicine educational course (19.1%) had a higher knowledge score (p-value <0.001). There was a significant association between the knowledge score on one hand, and the confidence level of respondents (p-value of 0.003) and their willingness to enroll in a disaster medicine course on the other hand (p-values <0.001).

**Conclusion:**

It is evident that medical students in Lebanon possess a fair level of knowledge and a high attitude towards disaster medicine.

## Introduction

Since the beginning of life, the world has been witnessing ongoing disasters, either natural or man-made. These disruptions exceed a country’s coping mechanisms and resources, thus affecting its functioning dramatically and harming its economy, infrastructure, and many more [1]. The healthcare field is one of the most affected systems at the time of a disaster in terms of the sudden increased need for medical attention to compensate in a relatively short time [2, 3]. Thus it is crucial for every community to be well-educated on this matter and fully prepared to manage the situation effectively when it arises [4].

In fact, the occurrence of disasters is increasing with time, as in the last century, the incidence of natural disasters in MENA (Middle East and North Africa) has remarkably increased, with around 212,832,485 affected people and nearly 614,000 deaths [5]. Recently, a series of massive earthquakes of high magnitude occurred in Turkey, resulting in extensive damage [6]. The recorded death toll surpassed 45,089, raising concerns about the potential outbreak of infectious diseases [6]. This series of earthquakes also hit Syria, where more than 6,000 deaths have been reported [6]. Lebanon has encountered several calamities as well. As an example, the natural wildfires that occurred in October 2019 were severe enough to destroy homes, evacuate people, and kill them [7]. Additionally, the man-made explosion that took place on August 4, 2020, in Beirut Port claimed 220 lives and caused around 6,000 injuries [8]. This blast also weakened the control of COVID-19, where, in the first week following, the number of positive cases increased to over 300 cases per day resulting in seven compared to a previous maximum of 100 cases per day. This situation significantly affected the majority of Beirut’s hospitals and medical centers [8, 9].

In light of these events, there has been an increased interest in the concept of disaster medicine. This medical discipline is concerned with the effects of natural or man-made disasters on human health. It covers the three stages of preparedness, reaction, and recovery, all of which seek to reduce the mortality and morbidity of affected communities [10, 11]. The best strategy to mitigate the consequences of a disaster is to provide adequate education about disaster medicine to a wide range of healthcare and medical professionals [12]. As the future generation of healthcare experts, medical students have the potential to offer valuable assistance in disaster medicine and therefore must be equipped with the knowledge and skills necessary to ensure an effective response to disaster [13–15].

In this context, many countries were found to have inadequate levels of knowledge about disaster medicine [12]. Based on previous studies conducted on healthcare personnel in developing countries, such as Yemen, Saudi Arabia, China, Ethiopia, and Malaysia, their knowledge and ability to help in case of a disaster were insufficient and somewhat acceptable [12]. In Italy, a group of 639 medical students participated in a Web-based survey, which revealed that 38.7% had never heard of disaster medicine, and 90.9% had not taken any elective academic courses on this subject [16]. Despite this, most of the participants expressed their willingness to have a course on disaster medicine included in their core curriculum and acknowledged its significance for their future [16]. Moreover, a research study conducted on medical students in Germany showed a significant increase in their level of knowledge after taking a disaster medicine course from 56% to 72%, with an increase in their level of interest in this topic from 63% to 80% [17].

To our concern, Lebanon is one of the developing countries where disaster medicine is not a part of the standard curriculum. Since 2013, the International Federation of Medical Students Associations (IFMSA) has conducted a training program on disaster medicine in many countries; Lebanon is one of them, indicating that some medical students are being taught and trained in this field [18], but to this day, no studies have been done to check their knowledge.

Hence, in this exploratory study, we aimed to examine medical students’ knowledge and attitudes toward disaster medicine in Lebanon, and how these varied by academic level, prior exposure to disaster-related education, and participation in life support courses. We anticipated that students with more advanced training and prior exposure would demonstrate higher knowledge scores and more positive attitudes.

In this study, knowledge refers to the student’s accurate understanding of disaster medicine concepts, such as disaster phases and the triage system. It was calculated as the number of correct answers to 25 multiple-choice questions. Attitude reflects students’ beliefs, opinions, and willingness to engage in disaster situations, and was assessed using a five-point Likert scale measuring agreement with statements related to disaster preparedness and training.

## Methods

### Study Design

An exploratory cross-sectional study was carried out from July 12 to August 22, 2023, among undergraduate medical students in Lebanon to assess their knowledge and attitude levels towards disaster medicine.

### Participants and Sample Size

The source population consisted of 3,580 undergraduate medical students registered during the 2022–2023 academic year from various universities in Lebanon. The inclusion criteria was all undergraduate medical students in Lebanon (both pre-clerkship and clerkship students). Exclusion criteria included pre-med students and medical students studying outside Lebanon. The sample size was calculated using an online sample size calculator [19] with a 95% confidence level, 5% margin of error, and an assumed population proportion of 50% which provides the most conservative estimate. This approach was chosen because the study is exploratory and descriptive in nature, aiming to estimate knowledge and attitudes in the target population. This resulted in a minimum sample of 348 medical students. 10% was added to overcome the missed information, bringing the total sample size to 387 participants.

### Tool Development and Validation

The questionnaire was adapted and modified after a detailed review of relevant literature from previously published studies [20, 21]. It was divided into three primary sections. The initial section gathered demographic data. The second part included one self-assessment yes-no question and 25 multiple-choice questions aimed to assess the knowledge of medical students regarding the phases of disaster management, triage, mass casualty, command system, terminologies, and classification of disasters. This knowledge section was adapted from a study conducted in South Africa [20]. The third section was composed of a five-point Likert scale (strongly agree, agree, neutral, disagree, strongly disagree) designed to evaluate the attitude towards disaster medicine, which was adapted from a study carried out in China [21]. As the majority of medical schools in Lebanon use English as the language of instruction, the questionnaire was developed in that language.

A pilot study was then conducted with 30 medical students from different universities to assess clarity, comprehension, and completion time. Based on the feedback, minor adjustments were made to improve wording and structure. Internal consistency of the questionnaire was assessed using data from the full study sample (n = 388). The knowledge section yielded a Cronbach’s alpha of 0.682, while the attitude scale had a Cronbach’s alpha of 0.628. Both values reflect acceptable reliability for exploratory research.

### Data Collection

Convenience sampling method was used. The data was collected through an online survey-type questionnaire designed using “Google Forms”. The survey link was initially distributed via social media platforms (WhatsApp and Instagram) through student groups at the various medical faculties in Lebanon. Student representatives were contacted to help disseminate the survey within their institutions. The data collection period lasted for 6 weeks (from July 12 to August 22, 2023). Although the sampling method included elements of snowball sampling, the primary strategy relied on open, voluntary participation through repeated social media postings. The participants were informed about the aim of the study before filling out the form.

### Data Analysis

The data was entered and analyzed via IBM SPSS Statistics 28.0. Variables were classified as categorical (e.g., gender, academic year…) and continuous (e.g., age, knowledge, attitude score…). Continues variables were expressed as mean and standard deviation (SD) while categorical variables were presented as frequencies and percentages.

Shapiro-Wilk test was used to assess the normality and shows deviation from a normal distribution. However, visual methods including histograms, box plots and Q-Q plot revealed an approximately normal distribution. This approach is supported by Ghasemi and Zahediasl [22], who suggest that normality tests should be complemented by visual methods, and that parametric tests remain valid in large samples despite minor deviations from normality. Given these findings and considering the Central Limit Theorem, the use of parametric tests was considered.

Accordingly, independent samples-t-test was used to compare between two groups and one-way ANOVA for comparison involving more than two groups. A p-value <0.05 was considered statistically significant.

### Ethical Consideration

The study has been approved by the Institutional Review Board (IRB) at Beirut Arab University. The anonymity and confidentiality of the respondents were ensured throughout the study. The data was secured correctly and saved on the researcher’s personal computer. Each response was coded by an ID number to maintain confidentiality. No one, except the investigator, had access to the data.

## Results

### Univariate Analysis

#### Demographic Characteristics

From a total of 392 participants, 4 were excluded due to missed information. Thus, 388 responses were included. Of them, 55.2% were female and 44.8% were male. The mean age (SD) was 21.92 (1.87) years. Concerning their educational level, 61.3% of the participants were in the pre-clerkship phase, whereas 38.7% were in the clerkship phase. The responses were gathered from different medical universities in Lebanon: American University of Beirut (AUB, 10.1%), Beirut Arab University (BAU, 19.8%), Holy Spirit University of Kaslik (USEK, 5.9%), Lebanese American University (LAU, 7.7%), Lebanese University (LU, 21.9%), Saint Georgie University of Beirut (SGUB, 1.8%), Université Saint-Joseph de Beyrouth (USJ, 16%), and University of Balamand (UoB, 16.8%). Of all the responses, only 19.1% of the participants have received an educational course or training on disaster medicine mainly through non-governmental organizations (63.5%), followed by elective courses (24.3%), and then the internet (12.2%). A fair number of respondents have received at least one of these international courses: Basic Life Support (BLS, 49%), Advanced Cardiac Life Support (ACLS, 18.3%), Advanced Trauma Life Support (ATLS, 5.7%), Pediatric Advanced Life Support (PALS, 4.4%), and Neonatal Resuscitation Program (NRP, 6.7%). From all participants, only 7.2% think they have sufficient knowledge concerning disaster medicine (Table 1).

**TABLE 1:** Socio-demographic information of respondents (Lebanon, 2023).

Characteristics	Mean ± SD
Age (in years)	21.92 ± 1.87
Characteristics	Frequency (%)
Gender	
Female	214 (55.2)
Male	174 (44.8)
Educational level	
Pre-clerkship	238 (61.3)
Clerkship	150 (38.7)
University	
American University of Beirut (AUB)	39 (10.1)
Beirut Arab University (BAU)	77 (19.8)
Holy Spirit University of Kaslik (USEK)	23 (5.9)
Lebanese American University (LAU)	30 (7.7)
Lebanese University (LU)	85 (21.9)
Saint Georgie University of Beirut (SGUB)	7 (1.8)
Université Saint-Joseph de Beyrouth (USJ)	62 (16.0)
University of Balamand (UoB)	65 (16.8)
Have you ever received any educational course or training on disaster medicine?	
Yes	74 (19.1)
No	314 (80.9)
If you answer yes, where did you receive the educational course or training on disaster medicine? (N = 74)	
Elective course	18 (24.3)
Non-governmental organizations	47 (63.5)
Internet	9 (12.2)
Students who answered “yes” to taking each of these courses	
Basic Life Support (BLS)	190 (49)
Advanced Cardiac Life Support (ACLS)	71 (18.3)
Advanced Trauma Life Support (ATLS)	22 (5.7)
Pediatric Advanced Life Support (PALS)	17 (4.4)
Neonatal Resuscitation Program (NRP)	26 (6.7)
Do you have enough knowledge about disaster medicine?	
Yes	28 (7.2)
No	360 (92.8)

#### Knowledge Assessment

Table 2 lists 25 multiple-choice questions for knowledge assessment about disaster medicine with a mean (SD) of 12.19 (4.04) for the correct answers. A Supplementary Table is present at the end for more detailed results.

**TABLE 2:** Knowledge of medical students about disaster medicine (Lebanon, 2023).

Questions	True (%)	False (%)	Mean + SD
Q1	59 (15.2)	329 (84.8)	12.19 ± 4.04
Q2	172 (44.3)	216 (55.7)	
Q3	261 (67.3)	127 (32.7)	
Q4	72 (18.6)	316 (81.4)	
Q5	208 (53.6)	180 (46.4)	
Q6	226 (58.2)	162 (42.8)	
Q7	199 (51.3)	189 (48.7)	
Q8	124 (32)	264 (68)	
Q9	189 (48.7)	199 (51.3)	
Q10	199 (51.3)	189 (48.7)	
Q11	272 (70.1)	116 (29.9)	
Q12	162 (41.8)	226 (58.2)	
Q13	221 (57)	167 (43)	
Q14	165 (42.5)	223 (57.5)	
Q15	111 (28.6)	177 (71.4)	
Q16	157 (40.5)	231 (59.5)	
Q17	177 (45.6)	211 (54.4)	
Q18	213 (54.9)	175 (45.1)	
Q19	239 (61.6)	149 (38.4)	
Q20	252 (64.9)	136 (35.1)	
Q21	147 (37.9)	241 (62.1)	
Q22	268 (69.1)	120 (30.9)	
Q23	243 (62.6)	145 (37.4)	
Q24	286 (73.7)	102 (26.3)	
Q25	110 (28.4)	278 (71.6)	

#### Attitude Assessment

Table 3 shows the summary of respondents’ attitudes towards disaster medicine. The majority (71.1%) of the surveyed students express a lack of confidence when it comes to handling disaster situations. Also, 83.7% disagreed that there is enough awareness to address different types of conflicts. Regarding disaster medicine training, 86.8% think that they need more workshops and simulated training to be ready to deal with a disaster, 84.3% were willing to attend disaster medicine education incorporated in the undergraduate coursework, and 85.9% agreed to be more trained on providing patient-centered care under a disaster situation.

**TABLE 3:** Attitude of medical students towards disaster medicine (Lebanon, 2023).

Attitude Statement	Total disagree	Strongly disagree	Disagree	Neutral	Agree	Strongly agree	Total agree
I feel confident in my abilities as a medical student in a disaster situation.	276 (71.1)	144 (37.1)	132 (34)	67 (17.3)	32 (8.2)	13 (3.4)	45 (11.6)
There is enough awareness on ways to stand wars and other humanity and natural emergencies among undergraduate students in university/medical college.	325 (83.7)	179 (46.1)	146 (37.6)	32 (8.2)	26 (6.7)	5 (1.3)	31 (8)
I need more workshops about disaster medicine and simulated training to be ready to deal with a disaster.	19 (4.9)	7 (1.8)	12 (3.1)	32 (8.2)	96 (24.7)	241 (62.1)	337 (86.8)
I am willing to attend the disaster medicine education incorporated in the undergraduate coursework.	22 (5.7)	7 (1.8)	15 (3.9)	39 (10.1)	136 (35.1)	191 (49.2)	327 (84.3)
I need to be more trained on providing patient-centered care under a disaster situation.	23 (5.9)	7 (1.8)	16 (4.1)	32 (8.2)	110 (28.4)	223 (57.5)	333 (85.9)

### Bivariate Analysis

#### Association Between Knowledge and Socio-Demographics

Table 4 shows the association between knowledge score and demographic characteristics. No statistically significant values were observed for the age group, educational level, and gender. Conversely, the knowledge score varied between universities with a p-value <0.001, as well as receiving an educational course and training on disaster medicine with a p-value <0.001. In addition, medical students who took BLS and ACLS were shown to have more knowledge regarding disaster medicine with a p-value <0.001 and 0.011 respectively.

**TABLE 4:** Association between knowledge and socio-demographics of participants (Lebanon, 2023).

Variable	Knowledge score mean/25	P-value
Gender		
Female	12.14	0.767
Male	12.26	
Educational level		
Pre-clerkship	12.11	0.614
Clerkship	12.33	
University		
American University of Beirut (AUB)	12.56	<0.001
Beirut Arab University (BAU)	10.96	
Holy Spirit University of Kaslik (USEK)	16.13	
Lebanese American University (LAU)	14.6	
Lebanese University (LU)	11.47	
Saint Georgie University of Beirut (SGUB)	11.28	
Université Saint-Joseph de Beyrouth (USJ)	11.08	
University of Balamand (UoB)	13.05	
Have you ever received any educational course or training on disaster medicine?		
Yes	14.89	<0.001
No	11.57	
If you answer yes, where did you receive the educational course or training on disaster medicine? (N = 74)		
Internet-based	12.22	0.041
Non-Internet base	15.14	
Have you took Basic Life Support (BLS)?		
Yes	13	<0.001
No	11.42	
Have you took Advanced Cardiac Life Support (ACLS)?		
Yes	13.29	0.011
No	11.95	
Have you took Advanced Trauma Life Support (ATLS)?		
Yes	13.41	0.148
No	12.12	
Have you took Pediatric Advanced Life Support (PALS)?		
Yes	13.35	0.228
No	12.14	
Have you took Neonatal Resuscitation Program (NRP)?		
Yes	13	0.294
No	12.1	

**FIGURE 1:**
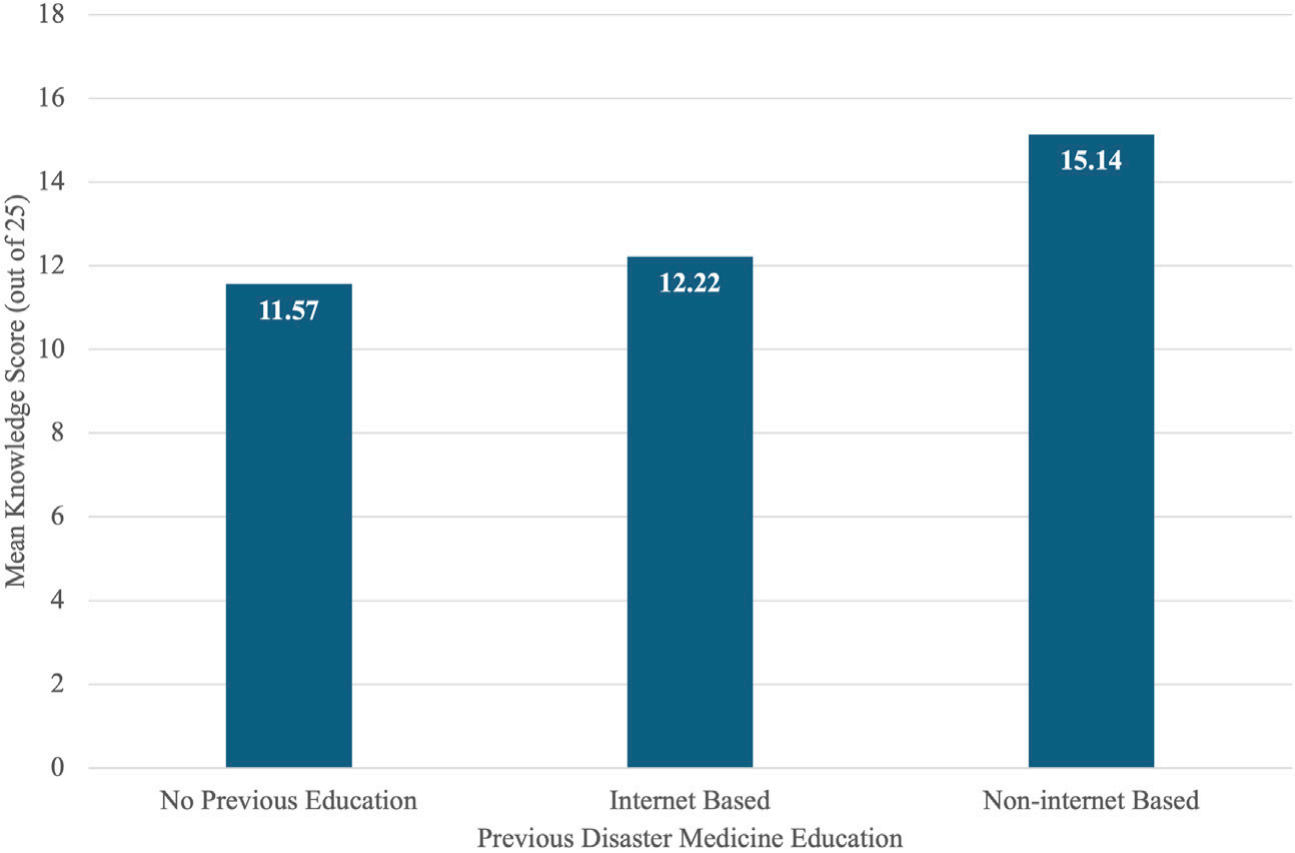
Mean disaster medicine knowledge scores by type of prior disaster medicine education among medical students in Lebanon (Lebanon, 2023).

Figure 1 presents the mean disaster medicine knowledge scores out of 25 by type of prior disaster medicine education. Students with no previous education had a mean score of 11.57. Those with internet-based education scored a mean of 12.22, while students with non-internet-based education had a mean score of 15.14.

**FIGURE 2:**
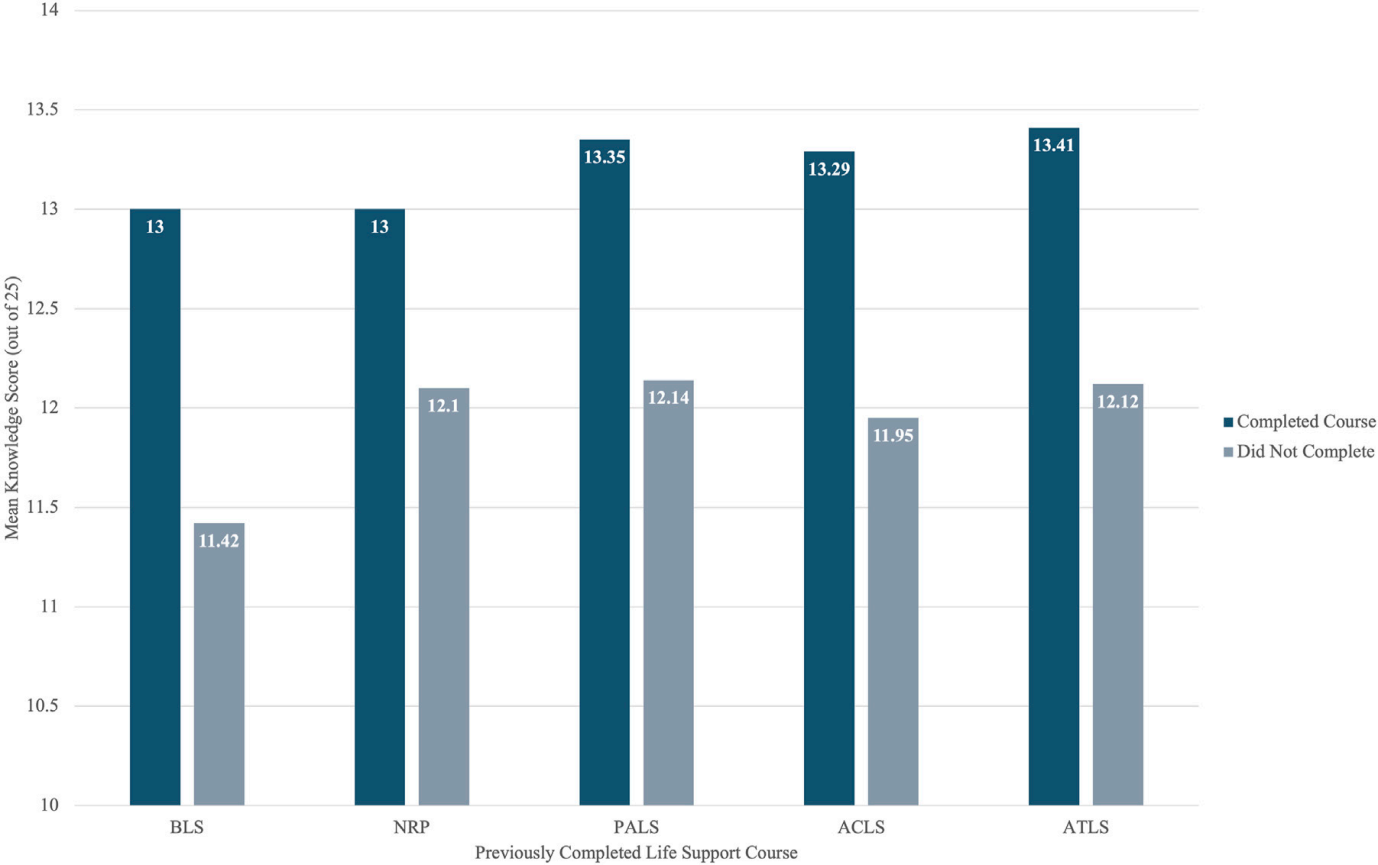
Mean disaster medicine knowledge scores by life support course completion among medical students in Lebanon (Lebanon, 2023).

Figure 2 illustrates the mean disaster medicine knowledge scores based on completion of various life support courses among medical students in Lebanon. Students who had completed BLS and NRP scored a mean of 13 each, while those who had not scored 11.42 and 12.1, respectively. For PALS, the mean score was 13.35 among completers versus 12.14 among non-completers. ACLS completers had a mean score of 13.29, compared to 11.95 for those who had not completed it. Similarly, the ATLS course showed a mean score of 13.41 among completers and 12.12 among those who did not complete it.

#### Association Between Knowledge and Attitude

Table 5 shows a significant association between the knowledge score and the confidence level and awareness when dealing with disaster situations, with a p-value of 0.003. Similarly, regarding disaster medicine training, there is a significant association between the knowledge score and the need for incorporating workshops, training, and a disaster medicine course in undergraduate education, with a p-value <0.001.

**TABLE 5:** Association between knowledge and attitude of participants (Lebanon, 2023).

Attitude Statement	Knowledge score mean/25	P-value
I feel confident in my abilities as a medical student in a disaster situation.		
Strongly Disagree	11.4	0.003
Disagree	12.62	
Neutral	13.49	
Agree	11.97	
Strongly Agree	10.61	
There is enough awareness on ways to stand wars and other humanity and natural emergencies among undergraduate students in university/medical college.		
Strongly Disagree	12.39	0.003
Disagree	12.73	
Neutral	10.69	
Agree	9.88	
Strongly Agree	11.2	
I need more workshops about disaster medicine and simulated training to be ready to deal with a disaster.		
Strongly Disagree	7.71	<0.001
Disagree	10.58	
Neutral	10.63	
Agree	11.73	
Strongly Agree	12.8	
I am willing to attend the disaster medicine education incorporated in the undergraduate coursework.		
Strongly Disagree	8.86	<0.001
Disagree	9.73	
Neutral	9.56	
Agree	12.5	
Strongly Agree	12.83	
I need to be more trained on providing patient-centered care under a disaster situation.		
Strongly Disagree	7.71	<0.001
Disagree	9.94	
Neutral	9.41	
Agree	12.29	
Strongly Agree	12.85	

### Multivariate Analysis

A multiple linear regression analysis was carried out among the 388 medical students to determine the factors influencing knowledge scores. The model was statistically significant, F (6, 381) = 10.500, p < 0.001, and explained 12.8% of the variance in knowledge scores (Adjusted R^2^ = 0.128). Greater age was significantly associated with higher knowledge scores (p = 0.006), as well as participation in at least one of the international courses (BLS, ACLS, PALS, NRP, ATLS) (p = 0.010). Medical students who received disaster medicine course primarily through non-internet-based methods demonstrated significantly higher knowledge scores (p < 0.001). Interestingly, clerkship students showed a significant lower knowledge scores compared to pre-clerkship students (p = 0.037), while there is non-significant association between gender or universities and knowledge score for p value = 0.897 and 0.831 respectively (Table 6).

**TABLE 6:** Multiple linear regression predicting disaster medicine knowledge score (Lebanon, 2023).

Predictor	B	SE	β	t	p-value
(Constant)	2.306	2.523	-	0.914	0.361
Gender (1 = Female, 2 = Male)	−0.051	0.392	−0.006	−0.129	0.897
Age (years)	0.336	0.122	0.156	2.747	0.006*
Educational Level (1 = Preclerkship, 2 = Clerkship)	−1.008	0.481	−0.122	−2.094	0.037*
University	0.024	0.112	0.011	0.213	0.831
Previous Disaster Medicine Education (1 = No, 2 = Internet Based, 3 = Non-internet Based)	1.581	0.274	0.294	5.780	<0.001*
Took at least 1 Life Support Course (BLS, ACLS, PALS, NRP, ATLS)	1.129	0.435	0.139	2.598	0.010*

Model statistics: F (6,381) = 10.55, p < 0.001; Adjusted R^2^ = 0.128.

Dependent Variable: Knowledge score.

B = unstandardized coefficient; β = standardized coefficient; SE = standard error.

## Discussion

This is the first study to assess the knowledge and attitudes of medical students from various universities in Lebanon regarding disaster medicine. Our findings revealed that the respondents have a fair level of knowledge in this area. Additionally, our results indicated that most medical students lacked confidence in handling disasters but acknowledged the need for simulated training to prepare for such situations. It is also important to highlight that there is a significant association between knowledge and attitude.

### Knowledge

Disaster medicine focuses on providing care to individuals affected by both natural and man-made disasters. It addresses the physical, emotional, and medical needs of those affected by disasters and includes disaster management. Therefore, future doctors need to be competent and willing to respond to disasters and to be involved in the areas of preparedness, recovery, and mitigation.

This study shows a significant difference in disaster medicine knowledge levels based on age, and educational levels with pre-clerkship students scoring higher than their counterparts. Similarly, Izquierdo-Condoy et al. not only found a significant association between knowledge level and both age and educational level but also reported gender difference [13]. This relationship contrasts with our study as gender level was not observed in our sample, possible due to more equal access to extracurricular training opportunities in disaster medicine across gender. Interestingly pre clerkship students scored higher, one possible explanation is the higher response rate from pre-clerkship students who might have been more involved in disaster medicine extracurricular workshops. In contrast, clerkship students may have more shifted focus towards clinical duties. This result is encouraging, as it suggests a growing interest and early engagement among younger medical students. Our study also showed no significant difference in knowledge between Lebanon’s medical universities after adjusting for all other variables. One explanation is the absence of a standard disaster medicine curriculum in Lebanese universities, meaning that students, regardless of gender and institutional affiliations begin with a similar knowledge base.

Although our study reveals that medical students possess a fair level of knowledge in disaster medicine, the mean knowledge score, which is 12.19 out of 25, is considered relatively low. Other studies conducted on Dutch and Singapore medical students showed an insufficient level of knowledge [23, 24]. This fair grade may be due to the recent introduction of this topic by national non-governmental organizations [18] and the contribution of the international life support courses to the medical students’ knowledge of disaster medicine. However, the low mean is primarily attributed to the absence of relevant curricula integration within Lebanese medical programs, hindering comprehensive preparation for such critical scenarios. Notably, our research shows that knowledge scores were higher in medical students who completed an educational course in disaster medicine, emphasizing how receiving such education enhances their knowledge. Similarly, Bajow et al. evaluated the effectiveness of a novel disaster medicine curriculum designed for undergraduate medical students in Saudi Arabia [25]. The study found that the curriculum effectively improved students’ knowledge of disaster medicine [25]. However, it is worth considering that this association may partly reflect self-selection by highly motivated students who may independently seek such courses or prior exposure to disaster-related volunteering.

As expected, medical students who completed international life support (BLS, ACLS, PALS, NRP, ATLS) courses showed statistically significant higher knowledge compared to their counterparts. This may be due to the overlap in time-critical decision making and developing response skills emphasized in both fields. Such courses utilize principles of experiential learning, as described in the Kolb’s cycle, where learners establish skills through repeated series of real-world practice, reflection, and conceptual integration—an approach ideally suited to the needs of disaster preparedness and response [26]. A study in Saudi Arabia supported this, indicating that those who took such courses had a higher knowledge level in emergency medicine [27]. This aligns logically with knowing that disaster medicine is an integral part of emergency medicine [28].

Interestingly, out of the 19.1% of students who took the course, only 7.2% stated that they had enough knowledge regarding disaster medicine. This inconsistency implies that participation in training does not necessarily result in perceived preparedness. A possible explanation is the discrepancy between factual knowledge and self-confidence; a pattern previously observed in disaster medicine education (Al-Hunaishi et al. [29]. Students become more aware of the scope and complexity of disaster response after structured education, leading to critical self-assessment and diminished confidence despite gains in knowledge.

### Attitude

The attitudes of medical students towards disaster medicine reflect a growing recognition of its importance in ensuring effective disaster response and management.

Based on our findings, the majority of respondents were not confident enough to handle and respond to disasters. Similarly, a study conducted in Latin America revealed that most of the participants did not believe in their abilities to act during disaster scenarios [13]. The fact that medical students showed a lack of knowledge and experience regarding disaster medicine is mainly due to both the absence of this field in their medical schools, the lack of training, and the limited education and awareness provided by the extracurricular programs. Contrary to our expectations, the findings reveal an association between elevated knowledge levels and reduced confidence, consistent with a study conducted in Turkey [30]. This may reflect a cognitive phenomenon called the '' Dunning-Kruger '' effect where High performers are more aware of complexities and limitations, which can result in under-confidence, while Low performers lack the knowledge to recognize their incompetence, leading to inflated confidence [31], or can be due to the lack of training. Therefore, incorporating simulated-based training by mimicking real-world disaster scenarios for students to apply their knowledge enhances their confidence and preparedness as showed in both studies by Cowling et al. [20] and Shannon [32]. Hence, this shows that knowledge alone is not sufficient to build confidence that pushes medical students to help during disasters. This can raise alertness about the need to improve their practical skills about disasters before encountering them. The importance of training is further supported by Cheng et al., who reported that 92.8% of students believed they could contribute more effectively during disasters if they received adequate training [24].

Furthermore, the participants showed a positive attitude towards participating in disaster medicine workshops and trainings. This result is aligned with a study conducted in Singapore where 93.1% agreed that training should be provided for medical students [24]. Also, Yemeni health professionals considered that interactive workshops or field exercises were more beneficial than only lectures and presentations [33]. This highlights the importance of hands-on training, simulation-based scenarios, and experiential learning to enhance confidence.

Indeed, our study highlights the importance of integrating disaster medicine courses into the undergraduate medical education. This finding aligns with arguments by Hoffmann and Muttarak who emphasize the necessity of formal education in promoting disaster preparedness [34]. They noted that early education and training not only enhance levels of knowledge and preparedness, but it also improves awareness and perception of the risk of a disaster and allow them to respond effectively and influences their behavior in such situations. In fact, medical students in our study were willing to have disaster medicine education incorporated into their undergraduate studies which demonstrate their commitment to the topic, eagerness to improve their understanding of disasters, and the importance of incorporating disaster course in the medical schools’ curriculum. This was echoed by Izquierdo-Condoy et al., who found that Latin American and Caribbean medical students similarly recognized the need to increase their knowledge in disaster medicine and considered the effectiveness of introducing specific courses into the medical curriculum [13]. While our study did not directly assess the effectiveness of simulation-based training, previous research suggests that the goal of disaster curricula should include preparing students for mass casualty events through practical exercises Gable et al. [35]. Authors like Cowling et al. and Joseph et al. emphasize that simulation-based training may enhance preparedness and response skills [20, 36]. This highlights the increasing recognition, in both student perspectives and prior research, of the value of experiential components in disaster medicine education aimed at enhancing preparedness and response capacities.

Remarkably, the willingness of the participants in our study to attend courses on disaster medicine in their undergraduate education, as well as workshops and trainings, was strongly associated with the knowledge score, suggesting that higher knowledge scores are linked to increased engagement and a desire for further education in the field.

Moreover, our data reveals that most participants agreed on the importance of delivering patient-centered care during disaster medicine; a finding that aligns with recent research demonstrating that person-centered approaches remain uphold even in resource-constrained disaster settings [37]. This may reflect a growing awareness among the future physicians about the importance of maintaining patient-centered values, even in challenging disaster scenarios.

### Limitations and Strengths

This study had certain limitations that must be considered. The data was obtained via an online survey which may introduce self-selection bias. Although the majority of medical students learn in English, it is still not their native language, thus some misunderstanding and lack of clarity may occur. Also, there was difficulty collecting an equal number of responses from each educational level. This may be due to the fact that the number of students in pre-clerkship is higher than that in clerkship. Additionally, one of the questionnaire items (perceived knowledge) was assessed using a binary yes/no format, which may limit the depth of interpretation. While it was included for descriptive purposes, it was not used in the bivariate or multivariate analyses to avoid overinterpretation.

Despite the above-mentioned limitations, our research is a novel and comprehensive study in the field. The main strength lies in being the first study done in Lebanon that assesses medical students’ knowledge and attitudes about disaster medicine. Another strength is its contribution to the development of medical school curricula by shedding light on areas that may require improvement.

### Conclusion

Medical students cannot prevent disasters, but they can help minimize their devastating consequences by being well-prepared to respond effectively. The findings of this study show that medical students in Lebanon exhibit fair level of knowledge in disaster medicine. Despite the knowledge gap, students display a positive attitude towards improving their knowledge through attending workshops and participating in courses related to disaster medicine. This recommends incorporating essential lessons into the undergraduate curriculum, organizing interactive workshops, and providing training sessions. Overall, ongoing efforts are needed to enhance medical education in Lebanon and equip students with the necessary knowledge and skills needed to face any unanticipated disasters.

This research opens the door for further studies to examine how implementing disaster medicine training programs within the undergraduate curriculum can enhance the knowledge level and attitude of medical students.
