# Supplementary material for: Knowledge and Attitude of Medical Students in Lebanon Towards Disaster Medicine
Source: Int J Public Health. 2025 Jul 28;70:1608095. doi: 10.3389/ijph.2025.1608095 (PMC12336067; doi:10.3389/ijph.2025.1608095)
Supplement: Supplementary file 1 [file Table1.pdf]

Supplementary Table. Knowledge assessment of medical students about disaster medicine (Lebanon, 2023).

Characteristics	Frequency (%)
1. During an adult mass casualty incident, the following triage score can be used:	
The Emergency Severity Index	206 (53.1)
South African Triage Score	55 (14.2)
JumpSTART	68 (17.5)
Sieve and Sort	59 (15.2)
2. What are the three phases of disaster management?	
Preparation, response, recovery	172 (44.3)
Evacuation, rebuilding, rebranding	57 (14.7)
Preparation, planning, perception	43 (11.1)
Planning, evacuation, recovery	116 (29.9)
3. A victim appears quite still when you get to them. You shake the victim and shout. They do not respond. What do you do next?	
Go to the next person	54 (13.9)
Open their airway	261 (67.3)
Shout again	58 (14.9)
Tag them dead	15 (3.9)
4. A victim is moaning. What do you do next?	
Count respirations for 15 seconds	111 (28.6)
Open their airway	101 (26)
Ask them why they are moaning	104 (26.8
Check their capillary refill	72 (18.6)
5. Which do you not do during triage?	
CPR	35 (9)
Intubate a patient who is gasping	72 (18.6)
Remove wet clothing	73 (18.8)
All of the above	208 (53.6)
6. The safety officer should not:	
Dictate to other services what to do to protect themselves	27 (7)
Ensure that all hazards are identified that may harm the rescuers	85 (21.9)
Let any member of staff onto the scene without appropriate PPE	226 (58.2)
Worry about the ambient temperature and weather conditions	50 (12.9)
7. All these statements concerning triage are true, EXCEPT:	
The aim of triage is to do the most for the most number of patients	61 (15.7)
Triage remains the sole responsibility of the EMS service	199 (51.3)

Triage is a dynamic process, and the patient's color code may be altered	80 (20.6)
It is important that one triage system is adopted so that all the relevant services use the same system	48 (12.4)

8. Pick the one true statement about the tiers of command:

Gold command is responsible for on-scene operational decisions	124 (32)
Bronze commanders are sectional heads responsible for operational teams	105 (27.1)
Silver commanders should ideally function as separate command structures	54 (13.9)
Gold command may involve international organizations	105 (27.1)

9. A major incident is defined as:

More than 100 casualties	86 (22.2)
An incident requiring military intervention	43 (11.1)
An incident caused by a natural disaster	70 (18)
An incident that overwhelms local resources	189 (48.7)

10. With regards to patient handover, the "M" in the MIST acronym stands for:

Mechanism of injury	199 (51.3)
My call sign	48 (12.4)
Male or female	39 (10.1)
Medical history	102 (26.3)

11. Which category do the walking wounded fit into initially?

Dead	9 (2.3)
Delayed	271 (70.1)
Immediate	61 (15.7)
Urgent	46 (11.9)

12. The following are initial tasks that the first unit on the scene should do, EXCEPT:

Make a thorough assessment of the situation	67 (17.3)
Wait for the most senior ranked officer to set up command	162 (41.8)
Assign arriving personnel to different positions with which they are familiar	72 (18.6)
Take responsibility for all functions of the Incident Command System	87 (22.4)

13. A victim has a capillary refill of 4 seconds. What do you do next?

Open their airway	59 (15.2)
Check capillary refill again	68 (17.5)
Tag immediate and treat for shock	221 (57)
Tag delayed and treat for shock	40 (10.3)

14. Choose the one incorrect statement pertaining to transport of casualties from the scene of a major incident:

It is a critical component that requires careful planning and coordination	68 (17.5)
Various organizations, such as a private EMS, may be able to transport patients and should take instructions only from their own control centers	165 (42.5)
Fire and law enforcement agencies may assist with the transport of patients out of the bronze zone	74 (19.1)
Patients triaged Green or P3 still require transportation to a health facility	81 (20.9)

15. As regards equipment requirement for a major incident, all of the following are true, EXCEPT:

Equipment pre-planning is paramount to an effective response	69 (17.8)
The wearing of PPE, while important, should not delay the response to getting to the patients	111 (28.6)
Checking of safety equipment remains the responsibility of the individual who will be wearing it	170 (43.8)
The type of equipment required may vary depending on the nature of the incident	38 (9.8)

16. During a pediatric mass casualty incident, the following triage score can be used:

The Emergency Severity Index	77 (19.8)
START	127 (32.7)
JumpSTART	157 (40.5)
South African Triage Score	27 (7)

17. Which is most important?

Your safety	177 (45.6)
The safety of the team	109 (28.1)
The victim's safety	65 (16.8)
Whether the victim lives or not	37 (9.5)

18. The phonetic alphabet:

Is local to South Africa	-
Uses words only and not numbers	86 (22.2)
Is an effective way to spell words over a radio	213 (54.9)
Cannot be used by the fire service	89 (22.9)

19. Triage labels should have the following characteristics:

Be highly visible	51 (13.1)
Be waterproof but still allow for the documentation of essential clinical notes on the label	63 (16.2)
Have a simple means whereby the label can be attached to the patient	35 (9)
All of the above	239 (61.6)

20. The surge capacity of a hospital refers to:

Capacity to coordinate the triage of patients effectively	67 (17.3)

The ability of a hospital to expand its normal services rapidly to meet the increase in demand	252 (64.9)
The ability of a hospital to discharge patients to lower levels of care	38 (9.8)
Capacity to coordinate resources through effective pre-planning	31 (8)
21. With regards to communication using the METHANE mnemonic, the "A" stands for:	
Access to scene	147 (37.9)
Ambulance required	123 (31.7)
Analgesia	52 (13.4)
Adult triage sieve score	66 (17)
22. Safety is paramount to ensure:	
Staff are prevented from getting infectious illness	61 (15.7)
Safety of self, scene and survivor	268 (69.1)
Biohazards are dispersed in the community	45 (11.6)
Staff are maximally utilized on the scene until they burn out	14 (3.6)
23. With regards to CSCATTT, the "A" stands for:	
Activity	51 (13.1)
Assessment	243 (62.6)
Ambulance	62 (16)
Authority	32 (8.2)
24. The following are examples of man-made disasters, EXCEPT:	
Flood	286 (73.7)
Train accident	40 (10.3)
Bomb blast	40 (10.3)
Oil spill	22 (5.7)
25. With regards to Command and Control, pick the most correct statement:	
It is the first priority	110 (28.4)
It is assumed by multiple individuals	116 (29.9)
Command is established in a bottom-up fashion	80 (20.6)
Horizontal communication is employed in each service	82 (21.1)
